# Remarkable Protective Effects of Nrf2-Mediated Antioxidant Enzymes and Tissue Specificity in Different Skeletal Muscles of Daurian Ground Squirrels Over the Torpor-Arousal Cycle

**DOI:** 10.3389/fphys.2019.01449

**Published:** 2019-11-22

**Authors:** Yanhong Wei, Jie Zhang, Xia Yan, Xin Peng, Shenhui Xu, Hui Chang, Huiping Wang, Yunfang Gao

**Affiliations:** ^1^Key Laboratory of Resource Biology and Biotechnology in Western China, Ministry of Education, College of Life Sciences, Northwest University, Xi’an, China; ^2^School of Basic Medical Sciences, Ningxia Medical University, Yinchuan, China

**Keywords:** hibernation, oxidative stress, antioxidant enzymes, Nrf2/Keap1 signaling pathway, skeletal muscle

## Abstract

Hibernating mammals experience conditions of extreme oxidative stress, such as fasting, muscle disuse, and repeated hypoxic ischemia-reperfusion, during the torpor-arousal cycle. Despite this, they experience little oxidative injury and are thus an interesting model of anti-oxidative damage. Thus, in the current study, we explored the levels and underlying mechanism of oxidative stress and antioxidant capacity in three skeletal muscles [slow-twitch soleus (SOL), fast-twitch extensor digitorum longus (EDL), and mixed gastrocnemius (GAS)] of Daurian ground squirrels (*Spermophilus dauricus*) during hibernation. Results showed that hydrogen peroxide content in the EDL and GAS decreased significantly during pre-hibernation (PRE) and late torpor (LT) compared to levels in the summer active (SA) group. Furthermore, relative to SA levels, malondialdehyde content decreased significantly during interbout arousal (IBA) and early torpor (ET) in all three skeletal muscles and decreased in the EDL and GAS during LT. Compared with the SA group, glutathione peroxidase 1 (GPx1) and catalase (CAT) protein expression in the SOL and superoxide dismutase 1 (SOD1) and SOD2 expression in the GAS increased significantly during the entire hibernation season. Furthermore, SOD1 in the IBA group and CAT and GPx1 in the ET and LT groups increased significantly in the EDL. The activities of most tested antioxidant enzymes were higher in the IBA group than in the LT group, whereas CAT remained highly active throughout the hibernation season in all three muscles. Nrf2 and p-Nrf2 protein levels were significantly elevated in the SOL and EDL during hibernation, and increased during the PRE, IBA, and ET states in the GAS. Thus, activation of the Nrf2/Keap1 antioxidant pathway resulted in the elimination of excess reactive oxygen species (ROS). Specifically, ROS levels were maintained at physiological levels by the up-regulation of antioxidant enzyme expression in skeletal muscles under oxidative stress during hibernation, thus preventing oxidative injury over the torpor-arousal cycle. Different antioxidant patterns and oxidative stress levels were also observed among the different skeletal muscles of hibernating Daurian ground squirrels.

## Introduction

Hibernating animals display specific adaptations and physiological functions suitable for the conditions experienced during the torpor-arousal period, i.e., low temperature, fasting, immobilization, and repeated hypoxic ischemia-reperfusion. Torpor involves the coordinated suppression of many physiological functions, including metabolic rate (to 1–5%), body temperature (Tb) (1–5°C), heart rate, blood flow, and respiration (Geiser, 2004; Storey, 2010). However, intermittent episodes of arousal (generally < 24 h) and euthermia are also experienced during torpor, involving the rapid reperfusion of oxygen and blood to all tissues (Carey et al., 2003; Storey, 2010). For example, oxygen consumption in the liver mitochondria of thirteen-lined ground squirrels (*Spermophilus tridecemlineatus*) increases dramatically during arousal (by 36-fold) compared with that during torpor (Muleme et al., 2006) and oxygen consumption in Arctic ground squirrels (*S. parryii*) increases significantly at different points during arousal in hibernation (Toien et al., 2001). The dramatic changes in physiological functions experienced by hibernators during the torpor-arousal cycle are similar to repeated ischemia-reperfusion (Ma et al., 2005; Ni and Storey, 2010). For non-hibernating animals (including humans), reduced blood perfusion can cause abnormal tissue and cellular metabolism, resulting in ischemic injury; furthermore, when blood reperfusion is restored, tissue and cellular damage can worsen as excessive free radicals can attack tissues and cells after they regain blood supply (Wang and Yin, 2013). As important hindlimb tissues, skeletal muscles perform many vital motor functions and are highly sensitive to ischemia (Blaisdell, 2002). Ischemia-reperfusion, with the massive production of reactive oxygen species (ROS), can damage the vascular endothelium system and increase the permeability of blood vessel walls. This can, in turn, lead to skeletal muscle edema, platelet adhesion and aggregation in microvessels, and deeper muscle color, and in severe cases, fibrosis, contracture, and necrosis of limbs as well as life-threatening failure of distant internal organs (such as the heart and kidney) (Carden and Granger, 2000; Kalyanaraman, 2013). Therefore, oxidative stress in skeletal muscles is a crucial area of research. Previous studies on non-hibernating animals have shown that an increase in ROS to the point where antioxidant scavenging capacity is exceeded is a significant cause of ischemia-reperfusion injury in skeletal muscles (Liu et al., 2007; Li and Zhang, 2015). In contrast, despite experiencing high oxidative stress during hibernation, including repeated ischemia-reperfusion, hibernating mammals exhibit no (or limited) evidence of oxidative injury, cellular stress, inflammation, or neuronal pathology (Ma et al., 2005). Therefore, understanding the antioxidant defense ability and regulation mechanism of skeletal muscles in hibernating animals is an important issue.

Reactive oxygen species are intermediate products of cellular metabolism and play important roles in maintaining intracellular homeostasis and signal transduction (Espinosa-Diez et al., 2015). Under conditions of excessive ROS, organismal endogenous antioxidant defenses are initiated to maintain redox homeostasis; however, if the accumulation of ROS exceeds the body’s antioxidant elimination capacity, oxidative damage can occur (Espinosa-Diez et al., 2015). Previous studies have found that malondialdehyde (MDA), a biomarker of lipid peroxidation, significantly increases in the skeletal muscles of non-hibernating rats (Lin et al., 2018) and in the serum and hindlimb skeletal muscles of non-hibernating rabbits following ischemia-reperfusion (Li et al., 2017; Zhang et al., 2018). Furthermore, the activities of multiple antioxidant enzymes, including superoxide dismutase (SOD), catalase (CAT), and glutathione peroxidase (GPx), are significantly reduced in the skeletal muscles of non-hibernating rabbits experiencing ischemia-reperfusion (Zhang et al., 2018), with changes also observed in apoptosis gene expression in skeletal muscle fibers (e.g., increased Bax and decreased Bcl-2 expression) (Li et al., 2017; Zong et al., 2017). The modulation of antioxidants during hibernation has been investigated extensively. Prior studies have found that the maximum rate of ROS production by mitochondria isolated from the skeletal muscles of hibernating thirteen-lined ground squirrels does not differ between torpor and interbout arousal (Brown et al., 2012). Furthermore, the expression of multiple antioxidant proteins, including SOD1, SOD2, CAT, and GPx, increases significantly in the skeletal muscles of European ground squirrels (*S. citellus*) (Vucetic et al., 2013) and thirteen-lined ground squirrels during torpor (Allan and Storey, 2012; Xu et al., 2013; Wu and Storey, 2014). Although such studies have explored the antioxidant defenses of skeletal muscles in hibernating animals, they have not distinguished the susceptibility of different types of skeletal muscles to oxidative stress. Based on proteomic analysis, our laboratory previously showed that the expression of SOD1 and CAT increases in the soleus (SOL) of Daurian ground squirrels (*S. dauricus*) in post-hibernation, indicating that the SOL exhibits a stress reaction and increased sensitivity to oxidants (Chang et al., 2016). In conclusion, existing research suggests that mammalian hibernators establish efficient antioxidant defense measures to cope with the challenge of oxidative stress during torpor-arousal cycles. Although skeletal muscle is an essential hindlimb tissue in animal movement, few studies have examined oxidative stress and antioxidant defense in the different types of skeletal muscles of hibernators, which is important for understanding the mechanisms of skeletal muscle weight and functional maintenance during hibernation.

Antioxidant defenses are regulated by many transcription factors, with the NF-E2-related factor 2 (Nrf2)/Kelch-like ECH-associated protein 1 (Keap1) signaling pathway recognized as one of the most important for antioxidant damage (Espinosa-Diez et al., 2015). Under normal circumstances, the Nrf2 protein and its inhibitor (Keap1) undergo cytoplasmic interaction and proteasomic degradation, which suppresses Nrf2 transcriptional activity (Sun et al., 2007). Under increased ROS production, however, proteasomic degradation is hampered. This initiates the release of Nrf2 from cytoplasmic Keap1/Nrf2 complexes, followed by nuclear translocation and accumulation as well as heterodimeric reactions with *cis*-regulatory elements located on the promoter regions of genes that encode defense proteins/enzymes (Bryan et al., 2013; Lushchak, 2014). Up-regulated antioxidant enzymes include SOD (Park and Rho, 2002), CAT, peroxidases (Lushchak, 2011), thioredoxin (Kim et al., 2001), glutathione-S-transferases (GST) (Chanas et al., 2002), and glutamate-cysteine ligase (GCL) (Lushchak, 2012). Previous studies have shown that Nrf2 protein expression is significantly higher in the gastrocnemius (GAS) muscle of rats under ischemia-reperfusion than under normal conditions, indicating that the body can resist ROS through the Nrf2/Keap1 pathway (Zong et al., 2017). As mentioned earlier, hibernators up-regulate the expression of antioxidant enzymes in skeletal muscles during hibernation (Allan and Storey, 2012; Vucetic et al., 2013); however, the mechanism of this up-regulation and whether it is related to the Nrf2/Keap1 antioxidant defense pathway remain unknown.

Skeletal muscles are also essential during interbout arousal due to their role in shivering thermogenesis, which helps the body return to euthermia (Dark and Miller, 1997). Theoretically, however, this activity may generate excessive ROS production due to the swift recovery of oxygen consumption and mitochondrial respiration during arousal (Ruuge et al., 1991; Hermes-Lima and Zenteno-Savin, 2002; Sanderson et al., 2013). Moreover, different types of muscle fibers exhibit differences in mitochondrial productivity, energy metabolism, and effects on the body. Therefore, it is important to understand the levels and underlying mechanism of oxidative stress and antioxidant capacity in different skeletal muscles of mammalian hibernators, particularly the effects of ischemia-reperfusion and antioxidant defense strategies during the torpor-arousal cycle. Here, we investigated how the antioxidant system in the skeletal muscles of Daurian ground squirrels responds to conditions experienced during the torpor-arousal cycle (e.g., low temperature, fasting, mechanical unloading, and repeated hypoxic ischemia-reperfusion). The SOL muscle consists of slow-twitch oxidative type fibers (i.e., myosin heavy-chain type I, MHC I) (Fu et al., 2016; Wei et al., 2018a), which contain a large number of mitochondria and rely on aerobic oxidation to generate energy. Conversely, the extensor digitorum longus (EDL) muscle consists of fast-twitch glycolytic type fibers (i.e., MHC II) (Fu et al., 2016; Wei et al., 2018a), which contain fewer mitochondria and rely on anaerobic glycolysis to provide energy. The mixed gastrocnemius (GAS) muscle consists of both oxidative and glycolytic type muscle fibers, and thus both aerobic and anaerobic functions (Fu et al., 2016; Wei et al., 2018a). Therefore, we examined the levels of ROS (H_2_O_2_ and MDA), protein expression and activity of multiple antioxidant enzymes (SODs, CAT, and GPx), and expression of the Nrf2/Keap1 signaling pathway in three different skeletal muscles (SOL, EDL, and GAS) of Daurian ground squirrels during different periods of the torpor-arousal cycle.

## Materials and Methods

### Animals and Groups

Adult Daurian ground squirrels were caught from the wild. Upon return to the laboratory, animals were maintained under standard conditions at 18–25°C and provided with food and water *ad libitum* until the end of October, as detailed in previous studies (Gao et al., 2012; Yang et al., 2014). Squirrel Tb was measured via thermal visual imaging (Fluke VT04 Visual IR Thermometer, Fluke, United States). All experiments and protocols were approved by the Laboratory Animal Care Committee of the China Ministry of Health.

**FIGURE 1:**
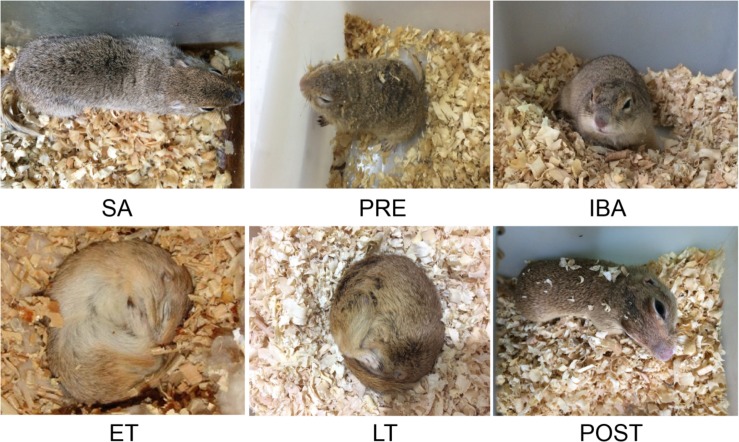
Images of Daurian ground squirrels during different hibernation periods. SA, summer active; PRE, pre-hibernation; ET, early torpor; LT, late torpor; IBA, interbout arousals; POST, post-hibernation.

Non-reproductive adult squirrels were weight-matched and randomly allocated into one of six groups (*n* = 8 in each group), following established experimental categories, i.e., summer active, pre-hibernation, interbout arousal, early torpor, late torpor, and post-hibernation groups (Wei et al., 2018b; Zhang et al., 2019). Details on the different states are listed in Supplementary Table S1 and Figure 1.

### Muscle Collection

**FIGURE 2:**
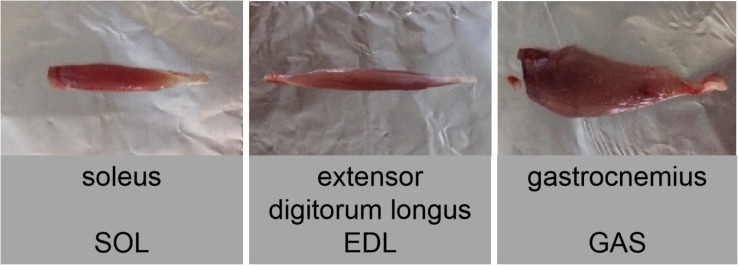
Images of skeletal muscles of Daurian ground squirrels.

For muscle collection, all animals were anesthetized with sodium pentobarbital at a dose of 90 mg/kg. Samples of the three hindlimb skeletal muscles (e.g., slow-twitch SOL, fast-twitch EDL, and mixed GAS) (Figure 2) were immediately removed, dissected, and weighed for determination of muscle wet weight, then subsequently frozen in liquid nitrogen and stored at −80°C until use. Upon completion of surgical intervention, all squirrels were euthanized with sodium pentobarbital via overdose injection.

### Quantification of H_2_O_2_ and MDA

As ROS are short-lived and highly reactive, their exact measurement in tissue samples remains difficult (Kohen and Nyska, 2002; Winterbourn, 2008; Kalyanaraman et al., 2012; Cheng et al., 2018). Here, the measurement of H_2_O_2_ (a significant ROS) and MDA (a secondary product) was used as an indicator for the levels of ROS.

Using a high-throughput tissue grinder (Scientz-48, Scientz Biotechnology, Zhejiang, China), frozen SOL, EDL, and GAS samples (∼0.1 g) were homogenized at 4°C in phosphate-buffered saline (PBS, 0.9 mL; containing 137 mM NaCl, 2.7 mM KCl, 10 mM Na_2_HPO_4_, and 2 mM KH_2_PO_4_). The tissue homogenates then underwent centrifugation (4°C, 15 min, 3000 rpm), with the protein concentration in the resulting supernatants determined using a Pierce^TM^ BCA protein quantitation kit (Thermo Fisher Scientific, Rockford, IL, United States) as per the manufacturer-provided instructions. The remaining supernatants were collected and kept on ice for further use in the following assays.

The concentrations of H_2_O_2_ and MDA in muscle samples were measured following Wei et al. (2018b) using H_2_O_2_ and MDA assay kits (Nanjing Jiancheng Bioengineering Institute, China), respectively, in accordance with the manufacturer’s protocols. The peroxo molybdate acid compound can act as a quantitative H_2_O_2_ indicator. Specifically, H_2_O_2_ can react with molybdic acid to form a stable peroxo molybdic acid compound, which exhibits maximum absorption at 405 nm. Therefore, the content of the compound can be measured at 405 nm via spectrophotometry (Shimadzu UV-2550, Kyoto, Japan). Muscle H_2_O_2_ content was then determined by comparing its OD_405_ value against those of the H_2_O_2_ standards.

As an index of oxidative damage, and the level of MDA can be used to indicate the level oxidative stress. Specifically, MDA readily reacts with thiobarbituric acid (TBA) to generate an MDA-TBA adduct (a type of thiobarbituric acid reactive substance, TBARS), which can be quantified colorimetrically. Here, the clarified supernatant derived from the skeletal muscle homogenate was mixed with the assay reagent containing TBA and butylated hydroxytoluene (BHT), with the latter used to reduce any artifactually formed lipid peroxides. The mixture was heated at 100°C for 40 min. After cooling, the mixture was centrifuged at 3000 rpm for 15 min at 4°C. The absorbance of the supernatants was then measured at 532 nm via spectrophotometry (Shimadzu UV-2550, Kyoto, Japan). Muscle MDA concentration was then determined by comparing its OD_532_ value against those of the MDA standards.

### Antioxidant Activity Assay

For the determination of antioxidant enzyme activity, frozen skeletal muscle tissues (∼0.1 g) were homogenized in ice-PBS (0.9 mL; containing 137 mM NaCl, 2.7 mM KCl, 10 mM Na_2_HPO_4_, and 2 mM KH_2_PO_4_) with a high-throughput tissue grinder (Scientz-48, Scientz Biotechnology, Zhejiang, China). The tissue homogenates then underwent centrifugation (4°C, 15 min, 3000 rpm), with the protein concentration in the resulting supernatants determined using a Pierce^TM^ BCA protein quantitation kit (Thermo Fisher Scientific, Rockford, IL, United States) as per the manufacturer-provided instructions. The remaining supernatants were kept on ice and used for the measurement of SOD, GPx, CAT, and TAC levels. In accordance with previous research (Wei et al., 2018b), antioxidant activity and total antioxidant capacity (TAC) in the muscle homogenates were ascertained using specific SOD, GPx, CAT, and TAC assay kits (Nanjing Jiancheng Bioengineering Institute, China) as per the manufacturer’s respective instructions, with experimental procedures and principles briefly described below. All antioxidant enzyme activities were measured at room temperature.

One unit of total SOD activity corresponded to a SOD inhibition rate of 50% per milligram of tissue protein in 1 ml of reaction solution (U/mgprot). One unit of CAT enzyme activity was defined as the amount of enzyme capable of directly decomposing H_2_O_2_, in a rate of 1 μmol/min, and one unit of GPx enzyme activity was defined as the amount of enzyme capable of directly consume substrate (GSH) in a rate of 1 μmol/min.

Superoxide dismutase activity was determined based on the auto-oxidation of hydroxylamine, followed by colorimetric (developed purple color) quantification at 550 nm. Briefly, the superoxide anion (O_2_^–^⋅) is produced by the xanthine and xanthine oxidase reaction, and O_2_^–^⋅oxidized hydroxylamine forms nitrite under the action of the color developer. When the sample to be tested contains SOD, it has a specific inhibitory effect on O_2_^–^⋅, which reduces the formation of nitrite. Under the colorimetric test, the absorbance value of the tube is lower than the absorbance value of the control tube, and calculation can be performed.

GPx can catalyze the reaction of H_2_O_2_ with reduced glutathione (GSH) to form H_2_O and oxidized glutathione (GSSG). If detection is carried out using H_2_O_2_ as a substrate, the enzymatic activity of catalase, which can also decompose H_2_O_2_, interferes with the measurement of GPx activity. We used an indirect assay here. The activity of GPx was determined by the speed of its enzymatic reaction and determined by the consumption of reduced GSH in this reaction. Moreover, the organic peroxide reagent (t-Bu-OOH) provided in the kit does not react with GSH in the absence of GPx, nor is it affected by the intracellular catalase catalytic and decomposed. Therefore, GSH peroxidase activity can be detected more specifically. In addition, because the two substrates can undergo a redox reaction in the absence of an enzyme, the portion of the reduced GSH produced by this non-enzymatic reaction must be subtracted when enzyme activity is finally calculated.

Catalase activity was determined by initially stopping H_2_O_2_ decomposition by CAT with ammonium molybdate, followed by colorimetric (developed pale-yellow color) quantification of the remaining H_2_O_2_ and ammonium molybdate complex at 405 nm via spectrophotometry (Shimadzu UV-2550, Kyoto, Japan).

For colorimetric determination, various antioxidants in an organism can reduce Fe^3+^ to Fe^2+^. Furthermore, Fe^2+^ can form a stable complex with phenanthroline, which exhibits strong absorption at 520 nm. Therefore, the level of antioxidant capacity can be measured colorimetrically.

### Quantitative of Total Protein

Ground squirrels change from glucose metabolism to lipid metabolism during hibernation. Thus, as a glucose-metabolizing enzyme, GAPDH expression is modified and inhibited during hibernation. For example, Bell et al. (2014) reported that GAPDH expression in the skeletal muscles of *Ictidomys tridecemlineatus* differs significantly between torpor and interbout arousal in regard to post-translational modification, phosphorylation, acetylation, and methylation. In jerboas (*Jaculus orientalis*), liver GAPDH relies on post-translational modification to inhibit activity during hibernation and decreases in GAPDH expression in skeletal muscle inhibit GAPDH activity (Soukri et al., 1996). Therefore, GAPDH cannot be used as an internal control reference. In addition, other internal reference proteins, such as α-tubulin, β-tubulin, and β-actin, are expressed differently in different tissues (Dittmer and Dittmer, 2006) and can (e.g., β-actin) vary with age in hibernators (Ferguson et al., 2005; Vigelso et al., 2015). However, total protein quantification can avoid these issues when used as a internal control in Western blot analysis (Aldridge et al., 2008; Ladner et al., 2004; Li and Shen, 2013). Therefore, we used total proteins in each group as the internal reference.

The electrophoresis separation gel was scanned with an electrophoresis gel imager (Syngene, United Kingdom), and the net optical density of the total proteins in each lane for all blots was determined using NIH Image J software.

### Western Blotting

We detected specific proteins in the tissue homogenates using Western blotting, as described previously (Wei et al., 2018a, b). Total proteins were extracted from the skeletal muscle samples using RIPA lysis (Heart, Xi’an, China). The protein concentrations were measured using a Pierce^TM^ bicinchoninic acid (BCA) protein quantitation kit (Thermo Fisher Scientific, Rockford, IL, United States), as per the provided instructions.

Equal volumes of proteins were used for 10% SDS-PAGE gel and polyvinylidene difluoride membrane blotting, as per prior research (Yang et al., 2014). After 45 min of electrophoresis, all proteins were transferred using 25-mM Tris buffer solution (pH = 8.5) to PVDF membranes (0.45 μm), with the membranes then blocked for 2 h with 5% fat-free milk and finally decanted. The membranes were subjected to overnight incubation (4°C) with specific primary antibodies for SOD1, SOD2, CAT, GPx1, Nrf2, Nrf2 (phospho S40), and Keap1 (as listed in Supplementary Table S2), followed by 2 h of incubation (room temperature) with HRP-linked anti-rabbit IgG secondary antibody (1:5000 v:v dilution, Thermo Fisher Scientific, United States) in TBST. The membranes were finally washed four times with TBST (5 min/wash) and visualized by enhanced chemiluminescence reagents (Thermo Fisher Scientific, United States) following the manufacturer’s instructions. Band intensity was determined using NIH ImageJ software. Total protein stain was applied for normalization to determine protein abundance (i.e., proteins of interest were normalized to total proteins in each lane for all blots) (Zhang et al., 2019).

### Statistical Analysis

Differences among experimental groups were assessed using one-way analysis of variance (ANOVA) and Fisher’s LSD *post hoc* tests. In cases of no homogeneity, ANOVA and Dunnett’s T3 tests were used. All statistical tests were conducted using SPSS v19.0 and a *P*-value of <0.05 was deemed significant.

## Results

### Changes in H_2_O_2_ and MDA Levels in Different Skeletal Muscles Over Torpor-Arousal Cycle

**FIGURE 3:**
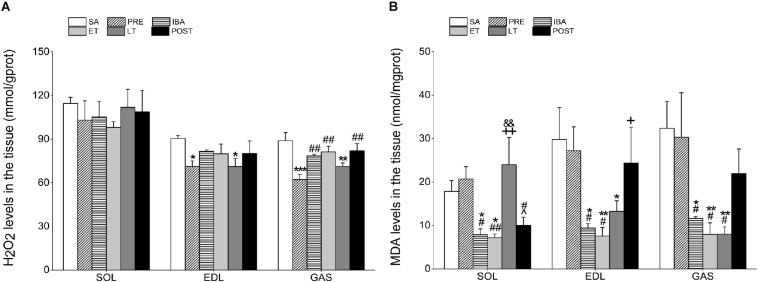
Levels of hydrogen peroxide (H_2_O_2_) and malondialdehyde (MDA) in different skeletal muscles. **(A)** Changes in H_2_O_2_ levels in SOL, EDL, and GAS during different periods (*n* = 8, one-way ANOVA). **(B)** Changes in MDA levels in SOL, EDL, and GAS during different periods (*n* = 8, one-way ANOVA). SA, summer active; PRE, pre-hibernation; IBA, interbout arousal; ET, early torpor; LT, late torpor; POST, post-hibernation. Data are means ± SEM. ^∗^*P* < 0.05, compared with SA; ^∗∗^*P* < 0.01, compared with SA; ^∗∗∗^*P* < 0.001, compared with SA; ^#^*P* < 0.05, compared with PRE; ^##^*P* < 0.01, compared with PRE; ^&^*P* < 0.05, compared with IBA; ^+^*P* < 0.05, compared with ET; ^++^*P* < 0.01, compared with ET.

As surrogate measures of oxidative stress and damage from free radicals, including ROS, we measured changes in MDA and H_2_O_2_ levels in the different skeletal muscles of hibernating ground squirrels (Figure 3).

Compared with the SA group, H_2_O_2_ content in the SOL showed no significant changes during the hibernation season. However, H_2_O_2_ levels in the EDL and GAS decreased significantly by 21.3% (*P* < 0.05) and 29.9% (*P* < 0.001), respectively, in the PRE group and by 21.4% (*P* < 0.05) and 19.8% (*P* < 0.01), respectively, in the LT group. In the POST group, H_2_O_2_ content recovered to SA levels in all three muscles (Figure 3A).

The MDA levels in all three muscles were markedly lower in the IBA and ET groups than in the SA and PRE groups. In the SOL, MDA levels in the LT group were significantly higher than those in the IBA and ET groups (204.8%, *P* < 0.01 and 233.1%, *P* < 0.01, respectively). However, MDA levels in the EDL and GAS muscles of the LT group decreased by 55.5% (*P* < 0.05) and 75.2% (*P* < 0.01), respectively, compared with the SA group. Similar to the changes in H_2_O_2_ levels, MDA content in all muscles recovered to normal levels after hibernation (Figure 3B).

### Expression of Antioxidant Proteins in Different Skeletal Muscles Over Torpor-Arousal Cycle

**FIGURE 4:**
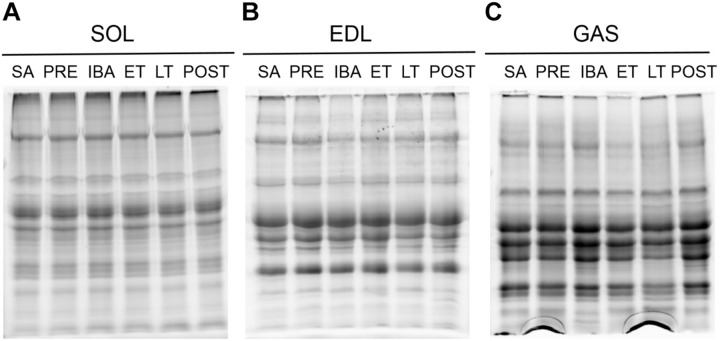
Stain-free imaging of separation gel. **(A)** In the SOL. **(B)** In the EDL. **(C)** In the GAS. SOL, soleus; EDL, extensor digitorum longus; GAS, gastrocnemius. SA, summer active; PRE, pre-hibernation; IBA, interbout arousal; ET, early torpor; LT, late torpor; POST, post-hibernation.

**FIGURE 5:**
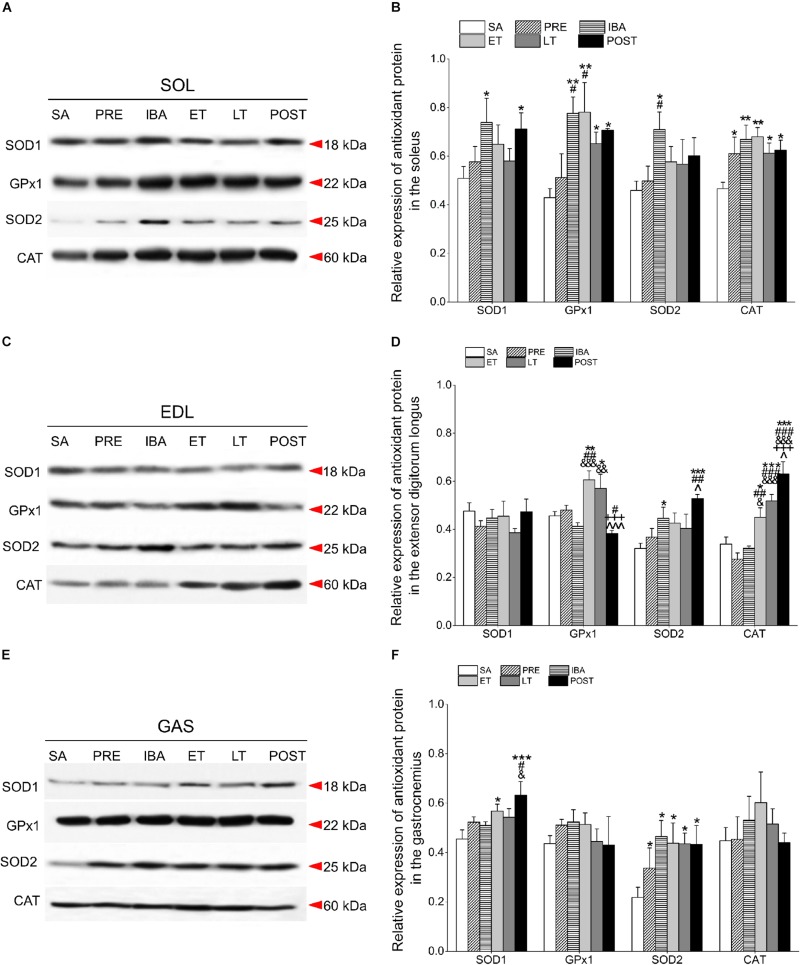
Changes in levels of SOD1, GPx1, SOD2, and CAT proteins in different skeletal muscles of Daurian ground squirrels over the torpor-arousal cycle. **(A)** Representative immunoblots of SOD1, GPx1, SOD2, and CAT in SOL during six hibernation periods. **(B)** Relative SOD1, GPx1, SOD2, and CAT protein expression in SOL. **(C)** Representative immunoblots of SOD1, GPx1, SOD2, and CAT in EDL during six hibernation periods. **(D)** Relative SOD1, GPx1, SOD2, and CAT protein expression in EDL. **(E)** Representative immunoblots of SOD1, GPx1, SOD2, and CAT in GAS during six hibernation periods. **(F)** Relative SOD1, GPx1, SOD2, and CAT protein expression in GAS. SA, summer active; PRE, pre-hibernation; IBA, interbout arousal; ET, early torpor; LT, late torpor; POST, post-hibernation. Values are means ± SEM, *n* = 8. ^∗^*P* < 0.05, Compared with SA; ^∗∗^*P* < 0.01, compared with SA; ^∗∗∗^*P* < 0.001, compared with SA; ^#^*P* < 0.05, compared with PRE; ^##^*P* < 0.01, compared with PRE; ^###^*P* < 0.001, compared with PRE; ^&^*P* < 0.05, compared with IBA; ^&⁣&^*P* < 0.01, compared with IBA; ^&⁣&⁣&^*P* < 0.001, compared with IBA; ^+++^*P* < 0.001, compared with ET; ^∧^*P* < 0.05, compared with LT; ^∧∧∧^*P* < 0.01, compared with LT.

The proteins of interest were normalized to total proteins (Zhang et al., 2019). Figure 4 shows the total protein gel images. In regard to antioxidant defense, we performed Western blot analysis to ascertain changes in the expression levels of several antioxidant proteins in the SOL, EDL, and GAS muscles, as shown in Figure 5.

Compared with the SA group, SOD1 expression in the SOL of the IBA and POST groups increased significantly (45.4 and 39.9%, *P* < 0.05, respectively). Furthermore, GPx1 expression remained at a higher level through the course of hibernation relative to the SA group, and increased by 81.0% (*P* < 0.01), 82.1% (*P* < 0.01), 51.8% (*P* < 0.05), and 64.7% (*P* < 0.05) in the IBA, ET, LT, and POST groups, respectively. SOD2 levels in the IBA group increased by 54.7% (*P* < 0.05). CAT protein remained at a high level throughout the hibernation season relative to the SA group, and increased by 30.9% (*P* < 0.05), 43.5% (*P* < 0.01), 45.9% (*P* < 0.01), 31.5% (*P* < 0.05), and 34.2% (*P* < 0.05) in the PRE, IBA, ET, LT, and POST groups, respectively. In the POST group, SOD1, GPx1, and CAT expression were higher, whereas SOD2 expression was similar to SA and PRE levels (Figures 5A,B).

Relative to the SA group, the SOD1 protein remained relatively stable in the EDL during hibernation. Furthermore, in the EDL, GPx1 expression increased significantly in the ET and LT groups (32.8%, *P* < 0.01; 25.0%, *P* < 0.05, respectively); SOD2 expression increased significantly in the IBA and POST groups (26.0%, *P* < 0.05; 64.9%, *P* < 0.001, respectively); and CAT expression increased dramatically in the ET, LT, and POST groups (32.8%, *P* < 0.05; 53.0%, *P* < 0.001; 86.0%, *P* < 0.001, respectively). In the POST group, SOD2 and CAT expression levels were higher, whereas SOD1 and GPx1 expression levels were similar to SA and PRE levels (Figures 5C,D).

In the GAS, SOD1 protein in the ET and POST groups increased markedly relative to the SA group (25.0%, *P* < 0.05; 39.1%, *P* < 0.001, respectively). In addition, SOD2 was at higher levels throughout the entire hibernation period, and increased by 54.3% (*P* < 0.05), 113.2% (*P* < 0.05), 100.6% (*P* < 0.05), 99.4% (*P* < 0.05), and 98.4% (*P* < 0.05) in the PRE, IBA, ET, LT, and POST squirrels, respectively. GPx1 and CAT expression in the GAS remained relatively steady during hibernation (Figures 5E,F).

### Antioxidant Enzyme Activity in Different Skeletal Muscles Over Torpor-Arousal Cycle

**FIGURE 6:**
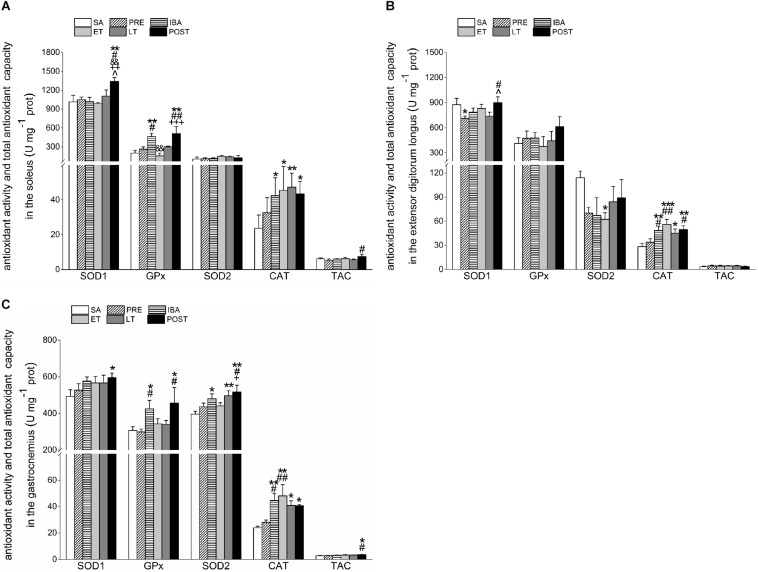
Changes in activities of SOD1, SOD2, CAT, GPx1, and TAC in different skeletal muscles of Daurian ground squirrels over the torpor-arousal cycle. **(A)** Activities of SOD1, SOD2, CAT, GPx, and TAC in SOL during six hibernation periods. **(B)** Activities of SOD1, SOD2, CAT, GPx, and TAC in EDL during six hibernation periods. **(C)** Activities of SOD1, SOD2, CAT, GPx, and TAC in GAS during six hibernation periods. SA, summer active; PRE, pre-hibernation; IBA, interbout arousal; ET, early torpor; LT, late torpor; POST, post-hibernation; SOL, soleus; EDL, extensor digitorum longus; GAS, gastrocnemius. Values are means ± SEM, *n* = 8. ^∗^*P* < 0.05, Compared with SA; ^∗∗^*P* < 0.01, compared with SA; ^∗∗∗^*P* < 0.001, compared with SA; ^#^*P* < 0.05, compared with PRE; ^##^*P* < 0.01, compared with PRE; ^&⁣&^*P* < 0.01, compared with IBA; ^+^*P* < 0.05, compared with ET; ^++^*P* < 0.01, compared with ET; ^+++^*P* < 0.001, compared with ET; ^∧^*P* < 0.05, compared with LT.

The activities of four antioxidant enzymes and TAC were determined to clarify their effects on oxidative stress, as shown in Figure 6.

Compared with the SA, PRE, IBA, ET, and LT groups, SOD1 activity in the SOL of the POST group increased significantly by 32.1% (*P* < 0.01), 27.7% (*P* < 0.05), 31.7% (*P* < 0.01), 35.9% (*P* < 0.01), and 21.3% (*P* < 0.05), respectively. Relative to the SA group, GPx activity increased significantly in the IBA and POST groups (130.1% and 153.8%, *P* < 0.01, respectively). Furthermore, SOD2 activity showed no obvious change in the SOL during the torpor-arousal cycle, whereas CAT activity increased markedly by 79.6% (*P* < 0.05), 92.2% (*P* < 0.05), 99.9% (*P* < 0.01), and 83.6% (*P* < 0.05) in the IBA, ET, LT and POST, respectively. The TAC in the SOL increased by 43.1% (*P* < 0.05) in the POST group compared with the PRE group (Figure 6A).

Compared with the SA group, SOD1 activity in the EDL decreased significantly by 18.8% (*P* < 0.05) in the PRE group, but recovered to SA levels in the POST group. GPx activity in the EDL did not show a clear change during hibernation, whereas SOD2 activity decreased throughout the hibernation season, with a significant decrease of 45.2% (*P* < 0.05) in the ET group. However, relative to the SA group, CAT activity in the EDL showed significant increases of 70.3% (*P* < 0.01), 97.0% (*P* < 0.001), 58.3% (*P* < 0.05), and 74.8% (*P* < 0.01) in the IBA, ET, LT and POST groups, respectively. The TAC in the EDL showed a slight but non-significant increase during hibernation (Figure 6B).

Compared with the SA group, SOD1 activity in the GAS increased throughout the hibernation season, with a significant increase of 20.7% (*P* < 0.05) in the POST group. Furthermore, GPx activity in the GAS increased significantly by 38.2% (*P* < 0.05) and 48.2% (*P* < 0.05) in the IBA and POST groups, respectively, and SOD2 activity increased significantly by 21.2% (*P* < 0.05), 25.5% (*P* < 0.01), and 30.7% (*P* < 0.01) in the IBA, LT, and POST groups, respectively. Relative to the SA group, CAT activity in the GAS increased considerably by 87.0% (*P* < 0.01), 101.5% (*P* < 0.01), 71.1% (*P* < 0.05), and 70.2% (*P* < 0.05) in the IBA, ET, LT, and POST, respectively. In addition, TAC in the GAS increased during the hibernation season, and increased markedly by 32.6% (*P* < 0.05) and 27.2% (*P* < 0.05) in the POST group compared with the SA and PRE groups, respectively (Figure 6C).

### Regulation of Antioxidant Transcription in Different Skeletal Muscles

**FIGURE 7:**
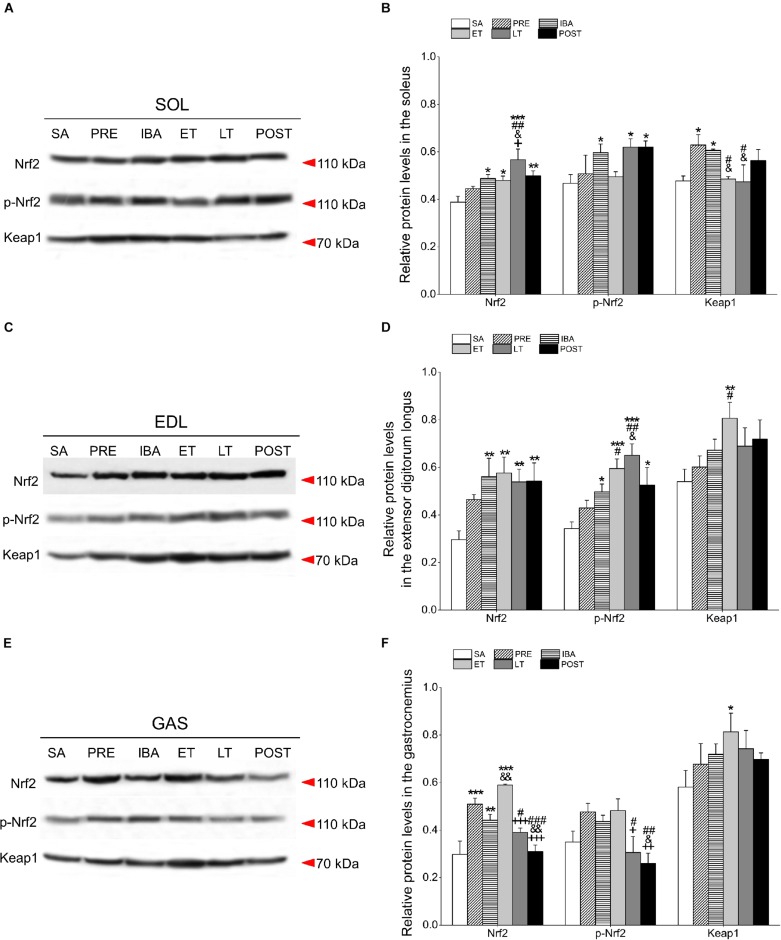
Changes in Nrf2, p-Nrf2, and Keap1 protein levels in different skeletal muscles of Daurian ground squirrels over the torpor-arousal cycle. **(A)** Representative immunoblots of Nrf2, p-Nrf2, and Keap1 in SOL during six hibernation periods. **(B)** Relative Nrf2, p-Nrf2, and Keap1 protein expression in SOL. **(C)** Representative immunoblots of Nrf2, p-Nrf2, and Keap1 in EDL during six hibernation periods. **(D)** Relative Nrf2, p-Nrf2, and Keap1 protein expression in EDL. **(E)** Representative immunoblots of Nrf2, p-Nrf2, and Keap1 in GAS during six hibernation periods. **(F)** Relative Nrf2, p-Nrf2, and Keap1 protein expression in GAS. SA, summer active; PRE, pre-hibernation; IBA, interbout arousal; ET, early torpor; LT, late torpor; POST, post-hibernation. Values are means ± SEM, *n* = 8. ^∗^*P* < 0.05, Compared with SA; ^∗∗^*P* < 0.01, compared with SA; ^∗∗∗^*P* < 0.01, compared with SA; ^#^*P* < 0.05, compared with PRE; ^##^*P* < 0.01, compared with PRE; ^###^*P* < 0.01, compared with PRE; ^&^*P* < 0.05, compared with IBA; ^&⁣&^*P* < 0.01, compared with IBA; ^+^*P* < 0.05, compared with ET; ^++^*P* < 0.01, compared with ET; ^+⁣++^*P* < 0.001, compared with ET.

The Nrf2/Keap1 signaling pathway is crucial for antioxidant defense regulation in hibernating mammals (Yin et al., 2016). In consideration of the effects of oxidative stress on this pathway during the torpor-arousal cycle, we measured Nrf2, p-Nrf2 (phosphorylated Nrf2), and Keap1 expression in skeletal muscles via Western blot analysis (Figure 7).

Compared with the SA group, the Nrf2 protein level in the SOL increased significantly by 25.7% (*P* < 0.05), 23.6% (*P* < 0.05), 46.2% (*P* < 0.001), and 28.6% (*P* < 0.05) in the IBA, ET, LT, and POST groups, respectively. Furthermore, relative to the SA group, the expression of p-Nrf2 in the SOL increased markedly by 27.8% (*P* < 0.05), 32.6% (*P* < 0.05), and 32.7% (*P* < 0.05) in the IBA, LT, and POST groups, respectively. Keap1 expression in the SOL increased by 31.8% (*P* < 0.05) and 27.1% (*P* < 0.05) in the PRE and IBA groups, respectively (Figures 7A,B).

Compared with the SA group, the Nrf2 protein level in the EDL increased significantly by 89.3% (*P* < 0.01), 94.7% (*P* < 0.01), 81.9% (*P* < 0.01), and 83.3% (*P* < 0.01) in the IBA, ET, LT, and POST groups, respectively. Furthermore, compared with the SA group, the p-Nrf2 expression in the EDL increased markedly by 45.4% (*P* < 0.05), 73.9% (*P* < 0.001), 90.1% (*P* < 0.001), and 21.3% (*P* < 0.05) in the IBA, ET, LT, and POST groups, respectively, and Keap1 expression increased by 49.5% (*P* < 0.01) in the ET group (Figures 7C,D).

Compared with the SA group, Nrf2 expression in the GAS increased significantly by 71.0% (*P* < 0.001), 48.6% (*P* < 0.01), and 98.1% (*P* < 0.01) in the PRE, IBA, and ET groups, respectively. The p-Nrf2 expression in the GAS showed a slight increase in the PRE, IBA and ET groups, whereas Keap1 expression showed a significant rising trend during hibernation, with a marked increase of 40.0% (*P* < 0.05) in the ET relative to the SA group (Figures 7E,F).

## Discussion

This study is the first to report on the remarkable protective effects of Nrf2-mediated antioxidant enzymes and tissue specificity in different skeletal muscles over the torpor-arousal cycle in Daurian ground squirrels. Results demonstrated that most tested indicators recovered to summer or pre-hibernation levels following their modification (increase or decrease) during hibernation, thus suggesting exceptional redox homeostasis. Moreover, the Nrf2/Keap1 signaling pathway was activated over the torpor-arousal period in the three skeletal muscles, which, in turn, up-regulated the expression of various antioxidant enzymes. These results indicated that the enhanced anti-oxidation defense ability mediated by the Nrf2/Keap1 pathway during repeated ischemia-reperfusion in skeletal muscles may be a vital mechanism to protect against oxidative damage during hibernation.

H_2_O_2_ is a significant ROS, and its increased production or failed breakdown can reflect increased oxidative stress (Halliwell and Cross, 1994; Turrens, 2003; Chainy et al., 2016). In the current study, H_2_O_2_ content in the EDL and GAS decreased significantly in the PRE and LT states. Pre-hibernation is a stage during which animals prepare for successful hibernation. For example, many hibernators will fatten extensively before the hibernation season to build-up fat stores that are relied on as an energy source during winter, or do not hibernate when lean (Geiser, 2013). Therefore, we speculated that this phenomenon may be the result of a forward-looking down-regulation of the oxidant system to cope with the multiple oxidative stresses experienced during hibernation; however, the specific mechanism is still unknown. Entrance into late torpor results in the transitory interruption of euthermic conditions, and includes considerable reductions in heart rate, blood flow, oxygen consumption, and mitochondrial respiration (Carey et al., 2003). For example, previous research has reported reductions in oxygen uptake of >90% in Arctic ground squirrels during late stage hibernation (Toien et al., 2001). Although animals face substantial stress during hibernation, including hypothermia, hypoxia, and ischemia, we found that H_2_O_2_ levels were decreased significantly in the LT group. This is similar to earlier studies on the visceral tissues of hibernators. For instance, Wei et al. (2018b) reported that H_2_O_2_ levels in the liver and kidneys of Daurian ground squirrels do not increase during hibernation. In addition, Yin et al. (2016), who studied the brain tissues of hibernating bats (*Myotis ricketti* and *Rhinolophus ferrumequinum*), demonstrated that ROS levels are lower during torpor than during the active state. In the current study, the significant decrease in H_2_O_2_ content in the EDL and GAS muscles during torpor suggests that oxidative stress did not occur in the skeletal muscles during hibernation, despite the stressful conditions. However, unlike the EDL and GAS, H_2_O_2_ levels in the SOL were slightly elevated in the LT state. One reason for this distinction may be the different proportions of muscle fiber type. The antigravity slow-twitch SOL muscle, which consists predominantly of MHC I oxidative fibers, contains a large number of mitochondria and relies on aerobic oxidation to generate energy. Conversely, fast-twitch EDL and mixed GAS muscles, which consist of MHC II glycolytic fibers or a mix of oxidative and glycolytic fibers, contain fewer mitochondria and rely on anaerobic glycolysis to provide energy. As mitochondria are the major sites of ROS generation, this may explain the higher levels of H_2_O_2_ found in the SOL during the LT state compared with that found in the EDL and GAS. In conclusion, the predominant decrease in ROS levels in the skeletal muscles during hibernation indicates that hibernating ground squirrels avoided oxidative stress, instead demonstrating remarkable redox homeostasis ability under high stress environments.

As a bioindicator of oxidative damage, MDA can indirectly reflect the levels of both oxidative stress and damage. In the present study, MDA content in the SOL increased markedly in the LT state compared with that in the IBA state. For successful hibernation, stored polyunsaturated fatty acid (PUFA) content is elevated in lipids in order to preserve lipid fluidity under low Tb conditions (Frank, 1992). However, due to their carbon-carbon double bonds, PUFAs are highly prone to free radical attack and are readily oxidized, resulting in lipid peroxide radical generation (Gunstone, 1996), and here in the observed MDA increase in the SOL during hibernation. The SOL muscle is mitochondrially rich and reliant on aerobic oxidation, which is significant given the importance of the mitochondrial electron respiratory chain in ROS production. Thus, compared to other skeletal muscles, the SOL is more sensitive to ROS via hypoxic ischemia-reperfusion. This is supported by previous study on Daurian ground squirrels, which showed that MDA content increases significantly during late torpor in tissues with the highest blood flow and oxygen consumption (i.e., heart and brain) (Wei et al., 2018b). In contrast, however, we observed significantly lower MDA content in the EDL and GAS muscles during LT than during the SA state. This indicated that under multiple stress conditions, the EDL and GAS not only avoided oxidative stress during hibernation, but the levels were lower than that observed under normal SA conditions. Moreover, MDA content in the IBA state was markedly lower than levels observed in the SA state in all three muscles, suggesting that IBA is an important period for hibernating animals to down-regulate ROS and prevent hibernation-induced oxidative stress. Under certain stress conditions, ROS production can become unregulated, resulting in the oxidization of various biomolecules, such as carbohydrates, lipids, and DNA, as well as the impairment of cellular functions and the promotion of cellular death (Espinosa-Diez et al., 2015). In contrast, under normal conditions, ROS play significant physiological roles as essential “second messengers” and are vital in intracellular signaling and regulation (Valko et al., 2007; Circu and Aw, 2010). This, together with the relatively low H_2_O_2_ levels observed during hibernation in our study, suggests that ground squirrels maintain a basal level of ROS during hibernation that retains function but does not harm tissue.

Shivering thermogenesis in skeletal muscle, which helps return Tb to euthermic levels, can actually trigger oxidative stress due to blood oxygen reperfusion and subsequent production of ROS (Barja de Quiroga, 1992; Dark and Miller, 1997). As oxidative stress is associated with both an increase in the production of oxidizing species and a decrease in antioxidant defense capability within the body, we detected changes in the protein expression of multiple antioxidants (i.e., SOD1, SOD2, CAT, and GPx1) in skeletal muscles under various stages of hibernation. In the SOL, the SOD1 protein levels in the IBA and POST states and SOD2 protein level in the IBA state increased markedly. The initiation of IBA is associated with the resumption of those physiological functions suppressed during torpor, including the rapid increase in Tb (35–38°C) and tissue reperfusion of blood and oxygen. Furthermore, at the completion of hibernation, animals experience a return of normal physiological function (Carey et al., 2003; Storey, 2010). Previous research on thirteen-lined ground squirrels has shown that oxygen consumption is significantly higher at arousal than during torpor (Muleme et al., 2006) and liver mitochondrial respiration rapidly increases following its suppression during hibernation (Mathers et al., 2017). Here, the significantly increased SOD1 and SOD2 expression levels observed in the IBA and POST groups suggest a need for SOD proteins in the SOL during the rewarming period when Tb recovers to euthermic levels. However, GPx1 and CAT expression levels in the SOL increased dramatically throughout the hibernation season, suggesting that these two antioxidant enzymes may contribute to improved antioxidant defense and protect cells for long periods during hypometabolism. Based on proteomic analysis, previous research on the skeletal muscles of Daurian ground squirrels found that SOD1 and CAT increase significantly in post-hibernation (Chang et al., 2016). SOD1 catalyzes the dismutation of O_2_^–^⋅and eliminates free radicals harmful to biological systems, whereas CAT decomposes H_2_O_2_ into H_2_O and protects cells from H_2_O_2_ toxicity (Halliwell and Cross, 1994; Chainy et al., 2016). As a slow-twitch muscle rich in mitochondria and dependent on aerobic oxidation (Peter et al., 1972), the SOL appears to be protected against potential oxidative stress-induced damage by the up-regulation of antioxidant enzymes. In the EDL, GPx1 and CAT expression during ET and LT increased markedly. Compared with levels under normal euthermic conditions, many physiological functions undergo temporary suspension or reduction during torpor (ET and LT), including substantial falls in metabolic rate (to 1–5%), Tb (as low as 1–5°C), blood flow, and oxygen uptake (Geiser, 2004; Storey, 2010). In the current study, however, under conditions of fasting and physiological inhibition, the expression of CAT and GPx1 were up-regulated, further suggesting the importance of enhanced antioxidant defense in hibernators to cope with and protect cells in high stress environments. In contrast, the SOD2 level increased substantially in the IBA and POST groups, implying that SOD2 may play a major role during IBA with high consumption of oxygen. Previous studies have shown that CAT, GPx, and SOD1 increase significantly and SOD2 decreases significantly in the skeletal muscles (quadriceps, fast-twitch muscle) of European ground squirrels during torpor (Vucetic et al., 2013). However, the protein expression of SODs in the present study is inconsistent with that reported in quadriceps, which may be due to the different effects of muscle mitochondrial productivity and energy metabolism on organisms. In the GAS, SOD1 protein expression increased significantly in the ET and POST groups, with SOD2 expression remaining at a high level throughout the hibernation season, and GPx1 and CAT remaining unchanged. These results suggest that SODs play a vital role in protecting against oxidative damage in the GAS. Earlier research has reported that TAC in the GAS is higher during torpor (156%) than in summer-active animals, suggesting a potential mechanism for maintenance of muscle performance (James et al., 2013). Here, the activity of antioxidant enzymes also increased in the IBA and POST groups compared to the LT group in all three skeletal muscles. We speculate that this may be caused by the accelerated blood flow velocity and recovery of normal physiological indicators during the IBA and POST states than that during the LT state. In addition, markedly increased CAT activity was observed over the hibernation season in all three muscles. Thus, this increase in activity may be a common skeletal muscle mechanism in hibernators to prevent oxidative injury due to repeated ischemia-reperfusion and prolonged muscle disuse over the torpor-arousal cycle. Nevertheless, in the present study, SOD2 activity showed different changes in the three skeletal muscles. Specifically, SOD2 activity increased significantly in various periods (IBA, LT, and POST) in the GAS, but showed no significant changes in the SOL and decreased in the EDL. Thus, the importance of SOD in the three skeletal muscles may differ. SOD2 activity was only elevated in the GAS, suggesting its importance for antioxidant capacity in acute muscle performance. However, the specific mechanism involved needs further study. During torpor, an animal experiences marked, though temporary, suppression in oxygen delivery and mitochondrial respiration; however, both are promptly restored during interbout arousal and accompanied by rapid tissue reperfusion (Carey et al., 2003; Storey, 2010). In response to ischemia-reperfusion, adequate antioxidant defense is needed to sustain cell viability over weeks of torpor and to defend against potential cellular stress during the transition stages. Indeed, our data showed that the protein expression and activity of four antioxidants increased significantly during hibernation bouts. Earlier studies have also demonstrated that various small molecular weight antioxidants and enzymes increase dramatically during torpor (Eddy et al., 2005; Ohta et al., 2006; Okamoto et al., 2006; Morin and Storey, 2007; Morin et al., 2008; Rouble et al., 2014; Yin et al., 2016; Wei et al., 2018b). For example, Vucetic et al. (2013) and Wei et al. (2018b) reported significant increases in SOD, CAT, and GPx1 protein and activity levels in the heart, liver, brain, and kidney tissues of Daurian ground squirrels and in the quadricep tissues of European ground squirrels, respectively, during hibernation. Allan and Storey (2012) found significant increases in SOD2 protein expression in mixed skeletal muscles during early hibernation, which remained high throughout, and Wu and Storey (2014) also found increases in CAT mRNA expression in the skeletal muscles of thirteen-lined ground squirrels. In conclusion, based on our observed results, the significant decrease in H_2_O_2_ and MDA levels during hibernation may be due to the decomposition and elimination of excess ROS following the up-regulation of antioxidant protein expression and activity, thus suggesting the importance of this up-regulation in preventing skeletal muscle oxidative damage. In addition, the expression of different antioxidant enzymes showed muscle tissue specificity. This may be due to different oxygen consumption and sensitivity to oxidants as well as the physiological state of different muscles during hibernation. Overall, among the three muscles, antioxidant enzymes were up-regulated at different stages of hibernation, indicating that the skeletal muscle of Daurian ground squirrels can control oxidative stress induced by repeated ischemia-reperfusion and skeletal muscle disuse.

Thus, hibernators appear to possess a well-developed antioxidant defense system. Antioxidant responses can be promoted in a variety of ways, including activation of gene regulating pathways. For example, the Nrf2/Keap1 antioxidant pathway plays a critical role in cytoprotective responses to oxidative stress (Motohashi and Yamamoto, 2004; Baird and Dinkova-Kostova, 2011). Thus, we further examined the expression of the Nrf2/Keap1 antioxidant pathway in the different skeletal muscles. Results showed the Nrf2 and p-Nrf2 protein levels increased significantly in the SOL and EDL throughout the hibernation season, and dramatically increased in the GAS during the PRE, IBA, and ET states. These results are similar to those found in studies on adipose, heart, liver, brain, and kidney tissues in hibernators. For example, Xu et al. (2013) reported that Nrf2 protein expression is significantly higher in the skeletal muscle (Xu et al., 2013), brown adipose tissue, heart, and liver of 13-lined ground squirrels (Morin et al., 2008) during hibernation than during the summer-active state. Ni and Storey (2010) demonstrated that significant increases in Nrf2 transcription factor expression levels in adipose tissues of hibernating squirrels during torpor and arousal can provide enhanced antioxidant defense to counter oxidative stress. Our previous research also showed increased Nrf2 and p-Nrf2 protein levels in the heart, liver, brain, and kidney tissue of hibernating Daurian ground squirrels (Wei et al., 2018a). The Nrf2/Keap1 signaling pathway is activated during oxidative stress, which results in the release of Nrf2 from Keap1 and its translocation into the nucleus. Nrf2 and small Maf proteins then undergo heterodimerization and antioxidant response element (ARE)-binding in the nucleus, with the subsequent activation of various cytoprotective genes (Sun et al., 2007; Espinosa-Diez et al., 2015). In our study, the significant increase in Nrf2 protein during torpor suggests that the elevated levels of antioxidant proteins (SODs, CAT, GPx) observed in the skeletal muscles during hibernation may be achieved through activation of the Nrf2 signaling pathway. Therefore, enhancing antioxidant defense ability by up-regulating the Nrf2/Keap1 antioxidant signaling pathway may be a common mechanism for hibernators to protect skeletal muscle and major visceral tissues from oxidative stress and may be triggered as one of the first events after entering the torpor-arousal cycle. Thus, hibernators may pre-emptively increase antioxidant defense in preparation for hibernation in order to manage the prolonged and repeated torpor-arousal cycle and muscle atrophy induced by disuse, i.e., “Preparation for Oxidative Stress” (Giraud-Billoud et al., 2019), especially in the SOL and EDL. In the GAS, however, the Nrf2 signaling pathway was only activated during the PRE, IBA, and ET states, but not during the LT and POST states. In the GAS, Nrf2 increased significantly during the PRE period, but the level of downstream antioxidant enzymes did not change significantly. We speculated that this may be because the sensitivity of antioxidant enzymes in the GAS is low, the reaction is slow, and the changes in antioxidant enzymes have not yet appeared. Conversely, because of the low sensitivity of antioxidant enzymes in the GAS, the antioxidant defense signaling pathway needs to be initiated earlier. In conclusion, activation of the Nrf2/Keap1 antioxidant pathway up-regulated antioxidant enzymes to eliminate excess ROS, which may play a vital role in skeletal muscle antioxidant defense. Furthermore, different antioxidant patterns were observed among the different types of skeletal muscle in the Daurian ground squirrels during hibernation.

## Conclusion

Our study demonstrated that hibernating ground squirrels cope with multiple oxidative stress conditions during hibernation via up-regulation of the protein and activity levels of multiple antioxidant enzymes, thus resulting in lower ROS levels (lower H_2_O_2_ and MDA content) and prevention of oxidative damage. The elevated antioxidant defense capacity mediated by the Nrf2/Keap1 signaling pathway may be an important mechanism of resistance to ischemia-reperfusion and disuse-induced oxidative damage over the torpor-arousal cycle. In addition, IBA is an important period for hibernating animals to down-regulate ROS and prevent hibernation-induced oxidative stress. Under conditions of physiological suppression during hibernation, the expression levels of various antioxidant proteins were up-regulated, further suggesting that enhancement of antioxidant defense ability is crucial for coping with high stress environments and achieving successful hibernation. Furthermore, different antioxidant patterns and oxidative stress levels were observed among the different types of skeletal muscle in the ground squirrels during hibernation. The current study not only provides a new perspective for understanding the adaptation mechanisms of hibernating squirrels to distinct ecological environments, but also provides a theoretical basis for novel therapies to treat ischemia-reperfusion damage-related diseases. Furthermore, this research could also be used to better understand how to protect organs from repeated ischemia-reperfusion.

## Data Availability Statement

All datasets generated for this study are included in the article/Supplementary Material.

## Ethics Statement

The animal study was reviewed and approved by the Laboratory Animal Care Committee of the China Ministry of Health.

## Author Contributions

YW, JZ, and YG conceived and designed the experiments. YW, XY, and XP performed the experiments. YW, HW, SX, and HC analyzed the data. YW and YG wrote and revised the manuscript.

## Conflict of Interest

The authors declare that the research was conducted in the absence of any commercial or financial relationships that could be construed as a potential conflict of interest.
