# Supplementary material for: Remarkable Protective Effects of Nrf2-Mediated Antioxidant Enzymes and Tissue Specificity in Different Skeletal Muscles of Daurian Ground Squirrels Over the Torpor-Arousal Cycle
Source: Front Physiol. 2019 Nov 22;10:1449. doi: 10.3389/fphys.2019.01449 (PMC6883408; doi:10.3389/fphys.2019.01449)
Supplement: Supplementary file 1 [file Table_2.DOCX]

**S2 Table. Antibodies used in this study**

| **Protein Name** | **Product No.** | **Company** | **Polyclonal or Monoclonal** | **Dilution** | **Sample loading/ lane** |
| --- | --- | --- | --- | --- | --- |
| SOD1 | ab13498 | Abcam Corp. | Polyclonal | 1:5000 | 20 μl |
| GPx1 | ab22604 | Abcam Corp. | Polyclonal | 1:1000 | 20 μl |
| Nrf2 | ab137550 | Abcam Corp. | Polyclonal | 1:500 | 20 μl |
| Nrf2 (phospho S40) | ab76026 | Abcam Corp. | Monoclonal | 1:5000 | 20 μl |
| Keap1 | ab139729 | Abcam Corp. | Polyclonal | 1:1000 | 20 μl |
| SOD2 | 13141S | Cell Signaling Technology | Polyclonal | 1:1000 | 20 μl |
| CAT | 14097S | Cell Signaling Technology | Polyclonal | 1:1000 | 20 μl |
