# Supplementary material for: Remarkable Protective Effects of Nrf2-Mediated Antioxidant Enzymes and Tissue Specificity in Different Skeletal Muscles of Daurian Ground Squirrels Over the Torpor-Arousal Cycle
Source: Front Physiol. 2019 Nov 22;10:1449. doi: 10.3389/fphys.2019.01449 (PMC6883408; doi:10.3389/fphys.2019.01449)
Supplement: Supplementary file 2 [file Table_1.DOC]

**S1 Table**  Details of the different hibernation states used in current study

| **Groups** | **Sample time** | **Tb of ground squirrels** |
| --- | --- | --- |
| Summer active (SA) | Squirrels not under hibernation in mid-June | > 37 °C |
| Pre-hibernation (PRE) | Squirrels had not yet entered torpor in late September | > 37 °C |
| Interbout arousal (IBA) | Squirrels aroused spontaneously from a new hibernation session after two months hibernation (68 ± 7 d) | 34–37 °C for less than 12 h |
| Early torpor (ET) | Squirrels entered a new hibernation session after two months hibernation (69 ± 8 d) | 5–8 °C for less than 24 h |
| Late torpor (LT) | Squirrels entered a new hibernation session and exhibited continuous torpor after two months hibernation (70 ± 5 d) | at least 5 d with stable Tb = 5–8 °C |
| Post-hibernation (POST) | Squirrels fully and spontaneously awakened from hibernation (121 ± 14 d) in the following spring (March) | > 37 °C for 3 d |
